# Casimir forces out of thermal equilibrium near a superconducting transition

**DOI:** 10.1038/s41598-022-06866-5

**Published:** 2022-02-21

**Authors:** S. G. Castillo-López, R. Esquivel-Sirvent, G. Pirruccio, C. Villarreal

**Affiliations:** Instituto de Física, Universidad Nacional Autónoma de México, Apartado Postal 20364, Mexico, 01000 Mexico

**Keywords:** Optics and photonics, Physics

## Abstract

We present a comprehensive analysis of the out-of-equilibrium Casimir pressure between two high-$T_c$ superconducting plates, each kept at a different temperature. Two interaction regimes can be distinguished. While the zero-point energy dominates in the near field, thermal effects become important at large interplate separations causing a drop in the force’s magnitude compared with the usual thermal-equilibrium case. Our detailed calculations highlight the competing role played by propagating and evanescent modes. Moreover, as one of the plates undergoes the superconducting transition, we predict an abrupt change in the force for any plate distance, which has not been previously observed in other systems. The sensitivity of the dielectric function of the high-$T_c$ superconductors makes them ideal systems for a possible direct measurement of the out-of-equilibrium Casimir pressure.

## Introduction

Two neutral parallel plates separated by a vacuum gap *L* attract each other due to quantum and thermal fluctuations. The original derivation by Casimir^1^ assumed perfect conducting plates at zero temperature. The extension to plates made of arbitrary materials and at a finite temperature was developed by Lifshitz^2^ using the fluctuation-dissipation theorem and Rytov’s theory of thermally induced electromagnetic fields^3,4^. Lifshitz theory has been well established through extensive theoretical and experimental work^5^ between metals^6^, semiconductors^7,8^, phase/change materials^9,10^, topological insulators^11,12^, among others.

When both plates are at the same temperature, the Casimir force has a contribution from quantum fluctuations at $T=0$ and thermal fluctuations^13,14^. At room temperature, for small separations ($L< 1\,\upmu$m) quantum fluctuations are dominant. At larger separations $L>3\,\upmu$m the thermal fluctuations are important^14^. The experimental observation of the thermal Casimir force was verified in an experiment by Sushkov and collaborators^15^. The study of finite temperature corrections to the Casimir pressure entails the yet ongoing debate of whether the Drude or plasma models is better suited to describe the properties of the zero frequency p-polarized mode in metals. In a series of works^16–22^, Bimonte et al. proposed to elucidate this controversy by measuring variations to the Casimir pressure in a rigid superconducting (SC) cavity for temperatures in the neighborhood of the transition temperature, $T_c$. Below this temperature, SC materials drastically modify their reflectivity properties from normal to superconducting; therefore, a pronounced variation $\delta P$ in the Casimir pressure should ensue. In principle, this effect could be detected by use of the contemplated cavity, specifically designed to observe changes in the Casimir pressure along the SC transition. A novel alternative device is an on-chip platform developed by Norte et al.^23^. It consists of two microfabricated strings, coated with a SC material, that can be kept perfectly parallel at sub-$\upmu$m separations and in which one of the strings is coupled to an optomechanical cavity with a definite resonance frequency. The variation of the Casimir pressure at the SC transition should modify the string mutual distance with an accompanying change in the cavity resonance frequency. This procedure may yield an estimation of $\delta P$ with a minimal resolution of 6 mN/$\hbox {m}^2$. However, for the classical BCS superconductors the predicted effects turn out to be too tiny to be detected by this sort of device^22^. In that context, the use of high-$T_c$ superconductors at sub- and $\upmu$m separations has been recently discussed in Refs.^24,25^. In the case of a pair of plates coated with $\hbox {YBa}_2\hbox {Cu}_3\hbox {O}_{7-x}$ (YBCO) and transition temperature $T_c = 93$ K, the characteristic thermal and zero-point frequencies, $k_B T_c/\hbar$ and $\omega _0=c/2L$, are of the same order of magnitude ($10^{14} s^{-1}$) for plate separations $L \simeq 1 \upmu$m, rendering these materials as optimally suited to test thermal contributions to the Casimir effect. For example, in the case with $L \sim 0.6 \upmu$m, the predicted $\delta P$ has a magnitude of $3 \%$ of the equilibrium pressure at $T_c$, $P^{eq}_{th} \approx 4$ mN/$\hbox {m}^2$. This quantity is nearby the resolution border of the experimental setup discussed above, and perhaps could be investigated through future improvements of this or similar devices. An alternative approach put forth by Bimonte is based on differential measurements, which offer the advantage of a much higher sensitivity in comparison to absolute force measurements^22^.

The problem of calculating the force when the two plates are at different temperatures was originally addressed by Dorofeyev using fluctuation electrodynamics^26^. A general theory of out-of-equilibrium Casimir and Casimir-Polder forces was presented by Antezza et al.^27–29^. The success of this latter formulation was shown by comparing with experimental measurements of the interaction between a Bose–Einstein condensate of trapped atoms and a flat surface at a temperature different from that of the environment^28^. Recently, the non-equilibrium Casimir force between two similar metallic plates of Au and Ti kept at different temperatures and considering the temperature dependence of their dielectric permittivity was calculated by Ingold et al.^30^. With that purpose, they introduced a temperature-dependent electronic relaxation rate, $\gamma (T)$. The derived results show a qualitatively different behavior than the case in which the dielectric function is independent of temperature. As mentioned by these authors, it will be of interest to study materials with a more sensitive temperature dependence.

In this work, we explore the nonequilibrium thermal effects on the Casimir pressure using high-temperature YBCO superconductors, whose dielectric function around the critical temperature changes drastically. With that purpose, we calculate the Casimir pressure between two parallel YBCO plates in contact with heat reservoirs at local temperatures, $T_1$ and $T_2$, separated by a vacuum gap of width *L* at zero temperature. We employ the formalism presented by Antezza^27^, taking into account the temperature dependence of the dielectric function around the superconducting phase transition. Similar methods based on the evaluation of the Poynting vector have been recently employed by us to evaluate the near-field radiative heat transfer between high-$T_c$ superconducting (HTSC) plates^31,32^. A central feature in that analysis is the role of evanescent surface plasmon modes, which may hybridize when the plates are close enough, inducing nontrivial features like the enhancement of the heat flux by thin SC films. In the context of the Casimir effect, Intravaia et al.^33^ have shown that in the zero-temperature limit, the Casimir energy of a dispersive cavity can be expressed as a sum over of electromagnetic modes of two different kinds: two evanescent surface plasmon mode arising from the p-polarization, and an infinite number of propagating modes due to p- and s-polarizations. Notably, the evanescent p contribution becomes repulsive for intermediate and large plate separations, although the total Casimir force remains attractive in any circumstance. In the following, we expand the former notions to study HTSC materials in a wide range of temperatures and surface separations, and investigate how the balance of evanescent and propagating contributions determines the behavior of the out-of-equilibrium Casimir pressure.

## Casimir force out of equilibrium

Consider a configuration out of thermal equilibrium constituted by two plates at local temperatures $T_1$ and $T_2$, separated by a gap *L*, and with optical properties described by a temperature-dependent dielectric function $\varepsilon _i(\omega ,T_i)$, with $i=1,2$. If we denote by $P^{eq}_{th}(T,L)$ the pressure associated with thermal fluctuations at equilibrium, then the total nonequilibrium Casimir pressure, $P^{neq}(T_1,T_2,L)$, may be expressed in terms of corrections $\Delta P(T,L)$ about the zero-point, $P_0(L{,T_1,T_2})$, and average equilibrium pressure, $\overline{P^{eq}_{th}}(T_1,T_2,L)=\left[ P^{eq}_{th}(T_1,L)+P^{eq}_{th}(T_2,L)\right] /2$^27,28^:

1 $$\begin{aligned} P^{neq}(T_1, T_2,L)= P_0({T_1,T_2},L)+\overline{P^{eq}_{th}}(T_1,T_2,L)+\ \Delta P(T_1,L)- \Delta P(T_2,L) -B(T_1,T_2), \end{aligned}$$

including the *L*-independent black-body term, $B(T_1,T_2)=2\sigma (T_1^4+T_2^4)/3$. In Eq. (1), the thermal *L*-dependent terms may be split into contributions due to propagating (PW) and evanescent waves (EW):

2a $$\begin{aligned} P^{eq}_{th,PW}(T,L)= & {} -\frac{\hbar }{\pi ^2}\int _0^\infty d\omega \ f(\omega ,T) \int _0^{\omega /c} dQ \ Q \ q_z\sum _{\alpha =s,p} \frac{ \left( r_1^\alpha r_2^\alpha e^{2i q_z L}\right) ^{\prime }-\vert r_1^\alpha r_2^\alpha \vert ^2 }{\vert D_{\alpha }\vert ^2}, \end{aligned}$$

2b $$\begin{aligned} P^{eq}_{th,EW}(T,L)= & {} \frac{\hbar }{\pi ^2}\int _0^\infty d\omega f(\omega ,T) \int _{\omega /c}^\infty dQ \ Q \ q_z^{\prime \prime } e^{-2 q_z^{\prime \prime } L} \sum _{\alpha =s,p} \frac{ \left( r_1^\alpha r_2^\alpha \right) ^{\prime \prime } }{\vert D_{\alpha }\vert ^2}. \end{aligned}$$

Here, $f(\omega ,T)=1/[\exp (\hbar \omega /k_B T)-1]$ is the Planckian distribution, *Q* and $q_z=q_z'+iq_z''=\sqrt{\omega ^2/c^2-Q^2}$ are the corresponding in- and out-plane components of the wavevector in the vacuum, and

3 $$\begin{aligned} D_\alpha (Q,\omega )=1-r_1^\alpha r_2^\alpha e^{2i q_zL}. \end{aligned}$$

Similarly, the out-of-equilibrium contributions are expressed as follows: $\Delta P(T,L)=\Delta P_{PW}(T,L)+\Delta P_{EW}(T,L),$ with

4a $$\begin{aligned} \Delta P_{PW}(T,L)= & {} -\frac{\hbar }{4\pi ^2}\int _0^\infty d\omega \ f(\omega ,T) \int _0^{\omega /c} dQ \ Q \ q_z \sum _{\alpha =s,p} \frac{\vert r_2^\alpha \vert ^2 - \vert r_1^\alpha \vert ^2}{\vert D_{\alpha }\vert ^2}, \end{aligned}$$

4b $$\begin{aligned} \Delta P_{EW}(T,L)= & {} \frac{\hbar }{2\pi ^2}\int _0^\infty d\omega f(\omega ,T) \int _{\omega /c}^\infty dQ \ Q \ q_z^{\prime \prime } e^{-2 q_z^{\prime \prime } L} \sum _{\alpha =s,p} \frac{r_1^{\alpha \prime \prime } r_2^{\alpha \prime } - r_2^{\alpha \prime \prime } r_1^{\alpha \prime }}{\vert D_{\alpha }\vert ^2}. \end{aligned}$$

In the above expressions, the reflection coefficients $r_i^\alpha$ correspond to $\alpha =\{\text{ s,p }\}$-polarized waves impinging on the (vacuum | *i*-plate) interface,

5 $$\begin{aligned} r_i^s=\frac{q_z-q_z^{(i)}}{q_z+q_z^{(i)}},\qquad r_i^p=\frac{\varepsilon _{i}(\omega ,T_i) q_z-q_z^{(i)}}{\varepsilon _{i}(\omega ,T_i)q_z+q_z^{(i)}}, \qquad i=1,2. \end{aligned}$$

Here, $q_z^{(i)}=\sqrt{\varepsilon _i(\omega ,T_i)\omega ^2/c^2-Q^2}$ represents the out-plane component of the wavevector inside the *i*-plate. Notice that in the case of similar materials with $r_1^\alpha = r_2^\alpha$, where $r_1^\alpha$ and $r_2^\alpha$ do not depend on temperature, the out-of-equilibrium contributions vanish, and the total pressure is given by $P_0({T_1,T_2},L)+\overline{P^{eq}_{th}}(T_1,T_2,L)$. However, since we are considering materials where the dielectric function $\varepsilon _{i}(\omega ,T_i)$ depends not only on the frequency of the incident light, but also on the (different) temperatures of the plates, $T_1$ and $T_2$, then the out-of-equilibrium terms yield a finite contribution that modify results proper of thermal equilibrium.

On the other hand, the zero-point contribution to $P^{neq}$ can be written, after a rotation to the imaginary frequency plane $\omega \rightarrow i\xi$, as follows:

6 $$\begin{aligned} P_0({T_1,T_2},L)=\frac{\hbar }{2\pi ^2c^3} \int _0^\infty d \xi \int _1^\infty \ dp \ p^2 \ \xi ^3\sum _{\alpha =s,p} \frac{r_1^\alpha (T_1) r_2^\alpha (T_2) e^{-2p\xi L/c}}{D_\alpha (Q,i\xi )}, \end{aligned}$$

with $p=\sqrt{1+c^2Q^2/\xi ^2}$. The zero-point term in Eq. (6) describes the action of zero-point radiation fields scattered by surfaces whose optical properties are described by temperature-dependent reflection coefficients, similarly as in the equilibrium case, where the reflection coefficients may depend, for example, upon the temperature by way of the electronic scattering rate, $\gamma (T)$. Due to convergence reasons, in this work the calculations involving the zero-point term have been performed in the imaginary frequency space, while those associated to thermal contributions have been performed in the real frequency space.

The dispersion relations of the allowed electromagnetic modes within the superconducting cavity, $\omega (Q)$, are determined by the zeros and branch cuts of Eq. (3). In the case of s-polarization, they admit an infinite number of PW modes. For p-polarization, besides the infinite PW modes, two-coupled evanescent fields arise, adopting either a symmetric or an anti-symmetric configuration, with respective dispersive relations $\omega _{-}(Q)$ and $\omega _{+}(Q)$. The development of this kind of low-frequency collective oscillations in the form of surface plasmons in both conventional and HTSCs is well established^34^. On the other hand, in the case of isolated surfaces (corresponding to the limit $L \rightarrow \infty$ of the cavity) a single evanescent field is generated about each one, with the corresponding relation, $\omega _\infty (Q)$, determined by the pole of the reflection coefficient, $r_p$. Explicit calculations show that, in general, $\omega _{-}(Q)<\omega _{\infty }(Q)<\omega _{+}(Q)$. It follows that, depending on the plate separation, the anti-symmetric mode may involve an energy excess over the infinite separation configuration, leading to a repulsive force. In contrast, the symmetric mode always induces an attractive force. Therefore, the detailed behavior of the Casimir pressure will be mainly determined the balance of the repulsive and attractive contributions for a given plate separation and temperature^33^.

In the calculations presented below, only *L* distance-dependent contributions are considered, so it was necessary to eliminate the *L*-independent term implicit in Eq. (4a)^28^.

## Optical response

YBCO is a highly anisotropic ceramic in which coherent charge transport mainly occurs along two $\hbox {CuO}_2$ planes (per crystallographic unit cell), denoted as *ab*-planes. In the optimally doped regime, superfluid transport is also observed to a small extent in the transverse *c*-axis direction. The optical response of uniaxial materials like YBCO is specified by a diagonal dielectric tensor diag$(\varepsilon _{ab},\varepsilon _{ab},\varepsilon _{c})$, whose components have been experimentally investigated for several compounds at different temperatures and frequencies using reflectivity and impedance-type measurements. Nevertheless, our explicit calculations show that the *c*-axis optical response yields negligible contributions in the evaluation of the Casimir pressure, and thus we neglect this contribution in the following.

In the normal regime at $T=100$ K, the dielectric function $\varepsilon _{ab}(\omega )$ has been represented as a superposition of a high-frequency term, $\varepsilon _{\infty }$, a Drude component due to free charge carriers, a mid-infrared (MIR) Lorentz contribution (maybe associated with indirect interband transitions), plus additional phonon contributions^35–38^:

7 $$\begin{aligned} \varepsilon _n(\omega ,T)=\ \varepsilon _{\infty }-\frac{\omega _{pn}^2}{\omega ^2 + i \gamma (T) \omega } - \frac{\Omega _{mir}^2}{\omega ^2 -\omega _{mir}^2 +i \Gamma _{mir} \omega } -\sum _{k=1}^{N_{ph}} \frac{S_{ph;k} \omega _{ph;k}^2}{\omega ^2-\omega _{ph;k}^2+ i\gamma _{ph;k} \omega }, \end{aligned}$$

where the plasma frequency $\omega _{pn}^2=e^2 {\rho _n} /\varepsilon _{0}m$. Here, ${\rho _n}$ is the number density of incoherent charge carriers with charge *e*, and mass *m*. On the other hand, the temperature-dependent electronic relaxation, $\gamma (T),$ has been fitted in this work to reproduce the observed linear dependence of the resistivity with temperature: $\gamma (T) = \gamma _0 + \beta T$, for $T>T_c$. Spectral measurements along the *ab*-plane may be fitted by the parameter choice: $\varepsilon _{\infty } = 3.8$, $\omega _{p}=1.14\times 10^{15}$ rad/s, $\gamma _{0}=4.56\times 10^{13}$ rad/s, $\beta =1.215 \times 10^{11}$ rad/s-K, $\Omega _{mir}=3.95\times 10^{15}$ rad/s, $\omega _{mir}=3.95\times 10^{14}$ rad/s, and $\Gamma _{mir} = 1.52\times 10^{15}$ rad/s. The number of optical phonons considered in the dielectric response is $N_{ph}=6$, and the parameters $S_{ph;ab,k}$, $\omega _{ph;k}$, and $\gamma _{ph;k}$, are presented in Ref.^31^.

A particularity of ceramic superconductors like YBCO arises from the combination of a strong oscillator strength $\Omega _{mir}$ of the MIR contribution with a large decay rate $\Gamma _{mir}$, which gives rise to a hybridization of the MIR modes with the low-frequency Drude modes. This mode coupling induces a surface plasmon with a frequency $\Omega _p\approx \left( (\omega _p^2+\Omega _{mir}^2)/\varepsilon _\infty \right) ^{1/2}\approx 1.6 \times 10^{15}$ rad/s, intermediate between $\omega _{p}$ and $\Omega _{mir}$; consequently, these materials display a metallic behavior for frequencies $\omega <\Omega _p$.

Corresponding measurements have been performed in the superconducting regime at $T=2$ K. In this case, the coherent charge transport has been modeled by considering the dissipationless limit $\gamma _0 \rightarrow 0$ in Eq. (7), which implies that $\omega _p^2/(\omega ^2 + i \gamma _{0} \omega ) \rightarrow \omega _p^2/\omega ^2 -i \pi \omega _p^2 \delta (\omega )/\omega$. However, an implicit assumption involved in this procedure is that all of the spectral weight of the normal-state Drude conductivity ends up under the zero-frequency delta distribution, which is valid only in the $T \ll T_c$ regime. Therefore, to characterize the YBCO optical response in the entire temperature interval, $0<T<T_c$, we introduce a temperature-dependent version of London’s two-fluid model of superconductivity. We suppose that, below $T_c$, the charge number density, ${\rho }$, may be split as follows: $\rho = \rho _n(T ) + \rho _s(T )$, where $\rho _n(T)$ and $\rho _s(T)$ correspond to normal and superfluid contributions. This allows the introduction of normal and superfluid plasma frequencies, respectively defined by $\omega _{pn}(T)=e^2 \rho _{n}(T) /\varepsilon _{0}m$, and $\omega _{ps}(T)=e^2 \rho _{s}(T) /\varepsilon _{0}m$. In that case, the dielectric response may be expressed as follows:

8 $$\begin{aligned} \varepsilon _s(\omega , T)= & {} \varepsilon _{\infty }+\frac{ i \pi \omega _{ps}(T)^2}{\omega } \delta (\omega )- \frac{\omega _{ps}(T)^2}{\omega ^2}-\frac{\omega _{pn}(T)^2}{\omega ^2 + i \gamma _{0} \omega } - \frac{\Omega _{mir}^2}{\omega ^2 -\omega _{mir}^2 +i \Gamma _{mir} \omega } \nonumber \\&-\sum _{k=1}^{N_{ph}} \frac{S_{mir;k} \omega _{ph;k}^2}{\omega ^2-\omega _{ph;k}^2+ i\gamma _{ph;k} \omega }. \end{aligned}$$

Here, $\omega _{ps}(2 \mathrm { K})=1.14\times 10^{15}$ rad/s, while the parameters $\varepsilon _{\infty }$, $\Omega _{mir}$, $\omega _{mir}$, and $\Gamma _{mir}$ are the identical to those considered in the normal phase, while the phonon parameters presented in Ref.^31^ are very similar as those involved in the normal case.

The charge transport properties of YBCO can be modeled in terms of a quasi-2D gas of pre-formed Cooper pairs that condense at temperatures $T\le T_c$, displaying a superfluid behavior. At relatively low temperatures, the condensate excitations may be accounted for by means of a gas of weakly-interacting bosons with a Bogoliubov dispersion law $E(k) = \sqrt{c_s^2 k^2+ (k^2/2m)^2 }$, where *E* is the energy, *k* the momentum, and $c_s$ the speed of sound. In the low momentum limit, this yields a phonon-like spectrum, $E (k) \approx c_s k$. A simple calculation shows that a 2D gas with this kind of dispersion relation satisfies the condition

9 $$\begin{aligned} \frac{\omega _{ps}^{2}(T)}{\omega _{ps}^{2}(0)} =\frac{{\rho _s}( T)}{{\rho _s}(0)} =1-\frac{T^2}{T_c^2}, \end{aligned}$$

which we substitute in Eq. (8) to describe the temperature dependence of the plasma frequency. Expression (9) yields an accurate representation of experimental measurements of $\omega _{ps}(T)=c/\lambda _p(T)$ (with $\lambda _p$ the magnetic penetration length and *c* light’s speed) along the $\hbox {CuO}_2$ plane for a wide range of dopings of $\hbox {YBa}_2\hbox {Cu}_3\hbox {O}_{7-\delta }$ samples^39–41^.

## Results

**Figure 1:**
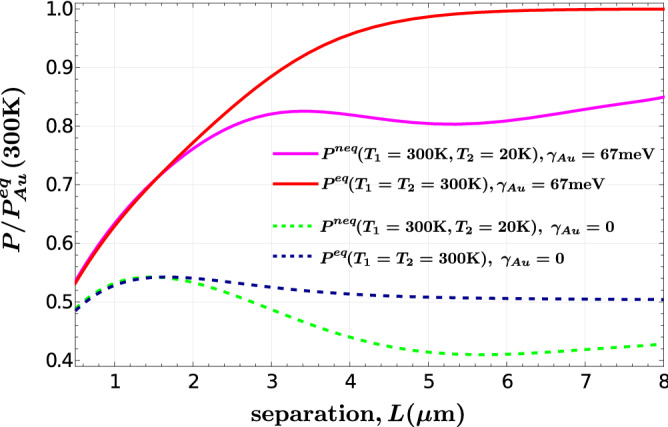
Casimir pressure between two semi-infinite YBCO plates in $(P^{eq})$ and out $(P^{neq})$ of thermal equilibrium as a function of the plates separation, *L*. The pressure axis is normalized to the equilibrium pressure between two gold plates at 300 K. When the gold permittivity is described using the Drude model with a finite damping rate $\gamma _{Au}=67$ meV, the normalization conduces to the pressure curves given by the solid lines. If the damping rate is neglected in the normalization $\gamma _{Au}=0$, the dashed lines are obtained.

We computed the Casimir pressure acting on two semi-infinite YBCO plates out of thermal equilibrium using Eq. (1) for temperatures above and below $T_c$. In order to compare with thermal equilibrium results, we also calculated the pressure provided by the Lifshitz expression at a fixed temperature $T_1=T_2$. In our first numerical experiment shown in Fig. 1, each plate is kept at different temperature, $T_1=300$ K and $T_2=20$ K, while the plate separation varies within the middle- to long-distance regime $L=0.5{-}8\,\upmu{\text{m}}$. Experimental investigations for normal metals have been carried out by Shushkov et al.^15^ in this distance scale. Also, we included the equilibrium Casimir pressure $P^{eq}$ obtained by setting $T_1=T_2=300$ K in Eq. (1). In order to gain insight on the magnitude of the predicted forces, we compare our results with those expected in the case of two gold plates in thermal equilibrium at 300 K. Then, the ordinate axis is normalized to the equilibrium Casimir pressure $P_{Au}^{eq}$ between two gold (Au) plates at 300 K: $P(T_1,T_2,L)/P_{Au}^{eq}(300\,{\text{K}},L)$. It turns out that the function $P(T_1,T_2,L)$ displays a monotonous *L*-dependence for temperatures far enough from $T_c$ (as considered in Fig. 1); this also occurs for $P_{Au}^{eq}(300\,{\text{K}}, L)$, but for all temperatures. Therefore, the pressure ratio shows a smooth behavior. However, in the Au case the question whether the Drude or plasma model should be applied to describe the Au permittivity becomes particularly relevant, since the absence or presence of the zero-frequency p-polarized electromagnetic contribution is determinant. We thus present our results by assuming both scenarios: The Drude model given by $\varepsilon _{Au}(\omega )=\varepsilon _{\infty ,Au}-\omega _{p,Au}^2/\omega (\omega +i\gamma _{Au})$, with $\varepsilon _{\infty ,Au}=9.84$, $\omega _{p,Au}=9.1$ eV, and $\gamma _{Au}=67$ meV, and the plasma model defined as the limit $\gamma _{Au} \rightarrow 0$ of the Drude permittivity. Figure 1 shows that in either case, both $P^{neq}(300\,{\text{K}}, 20\,{\text{K}}, L)$ and $P^{eq}(300\,{\text{K}}, L )$ attain values of the order of magnitude of the pressure acting between two gold plates. As expected, in the long-distance regime the predicted pressures in the plasma and Drude normalizations differ by a factor 1/2. Importantly, the equilibrium and nonequilibrium pressures may be clearly discriminated for plate separations $L>3\,\upmu$m, so that $P^{neq}(300\,{\text{K}}, 20\,{\text{K}}, L)$ is about $80\%$ of $P^{eq}(300\,{\text{K}}, L)$. Thus, the predicted decrease of the Casimir pressure as the system moves to a nonequilibrium situation seems feasible to be experimentally detected. We observe that the curves plotted in Fig. 1 display similar trends as those presented by Ingold et al. in Ref.^30^.

**Figure 2:**
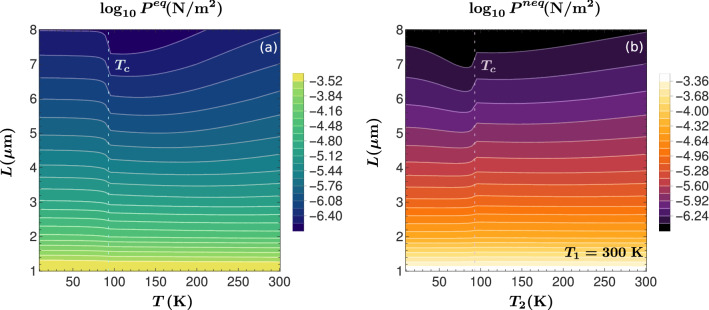
(**a**) Contour plot of the modulus of the equilibrium Casimir pressure (in logarithmic scale), $P^{eq}$, as a function of the separation distance, *L*, and the temperature of the YBCO plates, *T*. (**b**) Contour plot of the modulus of the nonequilibrium Casimir pressure (in logarithmic scale), $P^{neq}$, as a function of *L* and the temperature of plate-2, $T_2$, for a fixed temperature of plate-1, $T_1=300$ K.

For a complete analysis of the Casimir effect within the long-distance regime, we present contour graphs of the magnitude of the Casimir pressure as a function of the plate separation and the temperature. The contour lines in these figures represent isobaric trajectories along which the Casimir pressure is constant. Figure 2(a) corresponds to the equilibrium case in which the temperature of both YBCO plates is varied between 10 to 300 K. The nonequilibrium situation is presented in Fig. 2(b) where the temperature of plate-1 $T_1=300$ K, and the temperature of the second plate is swept within the range of 10–300 K. Consistently with results found in Ref.^24^, the equilibrium Casimir pressure shows a smooth behavior as a function of the separation distance, *L*. In contrast, its temperature dependence displays a sudden change in the slope at $T=T_c$, when both plates become superconducting, and the pressure suffers a noticeable increase for $T<T_c$, see Fig. 2(a). This manifestation of the SC phase transition results more evident for longer separation distances. On the other hand, the out-of-equilibrium pressure also exhibits a pronounced variation when the second plate passes to the superconducting state, $T_2=T_c$, as illustrated in Fig. 2(b). However, in this latter case the pressure suffers an abrupt decrease for temperatures below, but nearby $T_c$.

**Figure 3:**
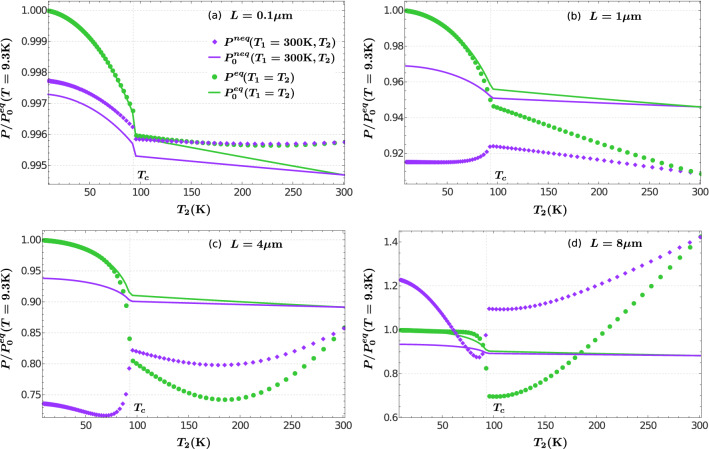
Temperature dependence of the equilibrium $P^{eq}$ and nonequilibrium $P^{neq}$ Casimir pressures for fixed separation distances of (**a**) $L=0.1\,\upmu$m, (**b**) $L=1\,\upmu$m, (**c**) $L=4\,\upmu$m, and (**d**) $L=8\,\upmu$m. The vertical axis of each figure is normalized to the corresponding zero-point pressure existing within two plates at 9.3 K: $P_0^{eq}(0.1\,\upmu{\text{m}})=2.95\,\hbox {N}/m^2$, $P_0^{eq}(1\,\upmu{\text{m}})=6.78\times 10^{-4}\,\hbox {N}/m^2$, $P_0^{eq}(4\,\upmu{\text{m}})=3.88\times 10^{-6}\,\hbox {N}/m^2$, and $P_0^{eq}(8\,\upmu{\text{m}})=2.72\times 10^{-7}\,\hbox {N}/m^2$.

In order to compare the contrasting trends of the Casimir pressure in- and out-of-thermal equilibrium, we plot in Fig. 3 the temperature dependence of $P^{neq}$ and $P^{eq}$ for four fixed separation distances of (a) $L=0.1\,\upmu$m, (b) $L=1\,\upmu$m, (c) $L=4\,\upmu$m, and (d) $L=8\,\upmu$m. In the figures below, the Casimir pressure is normalized to the zero-point contribution given by Eq. (6). For the short distance $L=0.1\,\upmu$m, Fig. 3(a) shows that the out-of-equilibrium corrections to the Casimir pressure are negligible since $P^{eq}\approx P^{neq}$ when the two YBCO plates are in the normal state. On the other hand, as the temperature decreases below $T_c$, both the equilibrium and nonequilibrium pressures increase abruptly. As mentioned above, within the long-distance regime $L=1{-}8\,\upmu$m, we observe different behaviors of $P^{neq}$ and $P^{eq}$ due to the superconducting phase transition. Figure 3(b) shows that while the equilibrium pressure between plates separated by $L=1\,\upmu$m suddenly increases below $T_c$, the nonequilibrium pressure shows a sharp decrease when plate-2 becomes superconducting. For temperatures both above and below $T_c$, $P^{eq}>P^{neq}$ at $L=1\,\upmu$m. When the plates are separated $L=4\,\upmu$m, Fig. 3(c), the nonequilibrium pressure becomes higher than the equilibrium one $P^{neq}>P^{eq}$ for a system made of two plates in the normal state. On the other hand, below $T_c$, $P^{neq}<P^{eq}$. At the last cut of $L=8\,\upmu$m, our calculations predict that $P^{neq}>P^{eq}$ except within the temperature range of 60–90 K, see Fig. 3(d). Additionally, each plot in Fig. 3 shows the zero-point contribution, $P_0^{eq}$ and $P_0^{neq}$, to the total Casimir pressure in- and out-of-thermal equilibrium (green and violet solid curves, respectively). In all cases, when the two YBCO plates are superconducting, the zero-point contribution $P_0^{eq}$ dominates the equilibrium pressure as $P_0^{eq}(T<T_c)\approx P^{eq}(T<T_c)$. In contrast, the nonequilibrium pressure differs from its corresponding zero-point contribution, $P_0^{neq}(T<T_c)$, because the thermal contribution coming from the plate in the normal state at $T_1=300$ K is still relevant.

**Figure 4:**
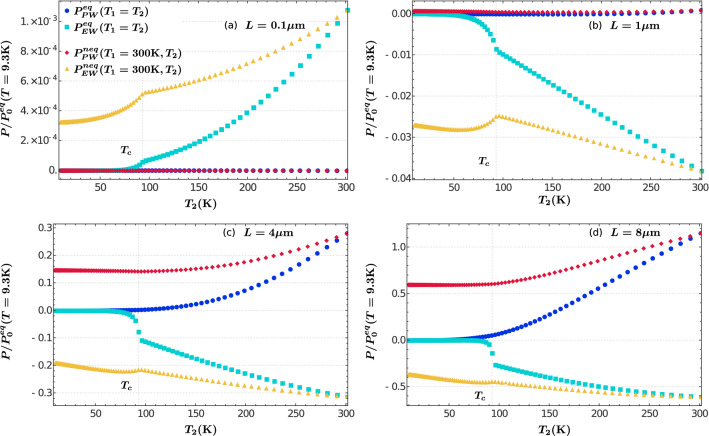
Temperature dependence of the thermal contributions to the Casimir pressure associated with EW and PW modes (according to Eqs. (2)–(4)), for the four cases of plate separation presented in Fig. 3. In each figure, the pressure axis is normalized to the corresponding zero-point contribution specified at the bottom of Fig. 3.

We analyze in Fig. 4 the thermal contributions to the Casimir pressure associated with EW and PW modes (according to Eqs. (2)–(4)) for the four cases described before. We observe that in the short-distance regime, $L=0.1 \upmu$m (Fig. 4a), the thermal contribution to the pressure arises solely from EW modes, both in equilibrium and out of equilibrium, yielding an attractive but negligible pressure of magnitude $\sim 10^{-4} P_0$, which increases with temperature. In fact, both equilibrium and out-of-equilibrium contributions coincide at $T \sim 300$ K. In contrast, PW modes provide a null contribution in the short-distance regime. Clearly, the EW contribution shows an abrupt change at $T_c$, decreasing its value for $T<T_c$. In particular, it becomes null in the equilibrium case, so that in this case only the zero-point term given by Eq. (6) yields a finite attractive force.

The sudden variation of the pressure at $T_c$ and the vanishing of the thermal contribution for $T<T_c$ at equilibrium is also displayed by EW modes at larger plate separations. However, as the separation increases towards the micrometer regime the associated pressure shows a shift from attractive to repulsive, see Fig. 4(b)–(d). Concomitantly, its relative magnitude increases not only with the separation but also with the temperature, so that at $L=8 \upmu$m and $T= 300$ K, it is about half the magnitude as $P_0^{eq}$. We notice that the structure of the EW contribution is very similar for $L \sim 1 \upmu$m. On the other hand, the influence of PW modes manifest itself at larger distances, giving rise to an attractive pressure that exceeds the repulsive action of the EW modes at all plate separations and temperatures.

## Conclusions

The Casimir pressure out of thermal equilibrium was analyzed between two high-$T_c$ superconducting plates kept at different temperatures, $T_1$ and $T_2$, for temperatures above and below the critical one, $T_c=93$ K. When the temperature of one of the plates varies in the range $T_2=10{-}300$ K, while the other is fixed at $T_1=300$ K, the former undergoes a superconducting transition at $T_2=T_c$ that manifests itself as a noticeable change in the slope of the nonequilibrium Casimir pressure, i.e. a discontinuity of its derivative. The abrupt variation of the pressure is also observed at thermal equilibrium, when the temperature of both plates changes within the same range $T_1=T_2=10{-}300$ K. However, in the long-distance regime $L=1{-}8\,\upmu$m, the equilibrium pressure increases abruptly below $T_c$, whereas the out-of-equilibrium pressure suffers a sharp decrease nearby $T_c$. The different behavior of the in- and out-of-equilibrium pressure is due to the thermal contribution of evanescent waves which show different temperature and separation dependences above and below $T_c$, but exhibiting in all cases a sudden change at $T_c$. In the near-distance regime, $L=0.1\,\upmu$m, these contributions are of attractive character, in contrast with the long-distance regime where they always provide a repulsive contribution proportional to the temperature. Of notice, the thermal contributions to the in-equilibrium pressure always vanish below $T_c$, with the zero-point terms being the only persisting ones. On the other hand, propagating modes always induce an attractive pressure whose influence becomes apparent at separations $L > 1 \upmu$m and balance the repulsion due to evanescent modes, yielding a net attractive Casimir pressure. The predicted equilibrium ($T_1=T_2$) and nonequilibrium ($T_1\ne T_2$) pressure turn out to be of the order of the Casimir pressure obtained experimentally between two gold plates at $T=300$ K. For distances $L>3\,\upmu$m, our results imply that nonequilibrium pressure is 80% of the equilibrium one, and they are consistent with those reported in Ref.^30^ for Au plates at different temperatures. The out-of-equilibrium Casimir force could be measured using the on-chip platform described before, based on an optomechanical cavity in combination with a grounded capacitor made of free-standing superconducting plates^23^. Our results suggest the feasibility of using high-$T_c$ superconductors to measure nonequilibrium effects on the Casimir pressure.
